# Microplate-based quantification of poly-γ-glutamic acid levels in biofilm samples

**DOI:** 10.1099/acmi.0.001162.v4

**Published:** 2026-06-09

**Authors:** David Stevenson, Cait E MacPhee, Nicola Stanley-Wall

**Affiliations:** 1 Division of Molecular Microbiology, School of Life Sciences, University of Dundee, Dundee, DD1 5EH, UK; 2 National Biofilms Innovation Centre, School of Physics and Astronomy, The University of Edinburgh, Edinburgh, EH9 3FD, UK

**Keywords:** *Bacillus subtilis*, microplate assay, poly gamma glutamic acid, quantification, standard curve

## Abstract

Poly-*γ*-glutamic acid (PGA) is a commercially useful biopolymer produced by many *Bacillus* species. PGA has a diverse range of applications across medicine and industry, generating significant interest in optimizing PGA production and enhancing yields. One approach to improve PGA recovery involves identifying high-yield PGA-producing strains and determining optimal production conditions, both of which require an appropriate screening method. Here, we present a sensitive and reproducible assay for quantifying PGA from *Bacillus subtilis* biofilms, whereby the spectral profile of methylene blue changes when bound to PGA. PGA was purified and lyophilized from NCIB 3610 Δ*tasA* liquid cultures grown at 50 °C, allowing production of protein-free PGA for use as a standard at known concentrations. Standard curves were generated from methylene blue absorbance readings at 564 and 664 nm, enabling subsequent quantification of PGA from biofilm extracts. We validated the quantification protocol and determined the treatment steps required to minimize interference. The assay has a 96-well plate format, enabling quantification of many samples at low sample volume, while minimizing waste of laboratory consumables. Overall, our method offers a sensitive, reproducible approach for PGA quantification in biofilm research and should facilitate comparative analyses across strains, treatments or environmental conditions.

## Graphical Abstract

Workflow illustrating poly-*γ*-glutamic acid (PGA) production, biofilm processing, protein level adjusted, SDS-PAGE analysis and colorimetric quantification, including controls and standards.

## Data Summary

Supplementary information is available on https://doi.org/10.15132/10000271.

## Introduction

Many *Bacillus* spp. produce a commercially relevant biopolymer known as poly gamma (*γ*) glutamic acid (hereafter PGA). PGA possesses several properties that are amenable to a wide variety of applications across many sectors [1,2]. Thus, PGA production has generated significant commercial interest. This is because PGA is biodegradable, non-toxic and non-immunogenic and avoids degradation by proteases (allowing possible applications in drug delivery); it is used in food and cosmetics and has been used for water purification steps [1]. However, a potential prohibitive drawback is that PGA is relatively expensive to produce and/or process. Production depends on the organism and growth or incubation conditions [3,6]. Attempts to reduce associated costs include an ongoing focus on finding or developing strains capable of producing high yields of PGA [1,7]. Therefore, improving screening for high-yield PGA producers could help lower production costs and make commercial ventures more viable. The search for higher yields necessitates the development of methods with enough sensitivity to distinguish between the strains of value for processing.

In *Bacillus subtilis*, PGA synthesis requires the *capBCAE* (*pgsBCAE*) operon [8], which encodes the enzymes responsible for polymerization of glutamic acid into PGA [9]. Our research focuses on biofilms, organized multicellular communities of microbes encased within a self-produced protective matrix [10]. For *Bacillus* spp., the matrix is composed of several key components, which contribute to structure and function and often include PGA [11,12]. Methods visualizing PGA have used polyacrylamide gels as a relatively quick method to gauge gross scale differences in PGA recovered under different conditions [4]. Here, we adapt work by Chatterjee *et al*., to develop a microplate-based PGA quantification assay utilizing methylene blue, based on a shift in absorbance readings for methylene blue when bound to PGA [13]. The microplate format allows many samples to be screened under a variety of conditions in a relatively quick and cost-effective manner, requiring less material and reagents and reducing waste.

## Methods

### Bacterial strains and growth conditions

*B. subtilis* NCIB 3610 (wild-type), NCIB 3610 Δ*tasA* (used for purification of PGA for standards) and NCIB 3610 Δ*capB* (PGA-negative control) were used in this study. Strains were maintained on Luria Broth (LB) agar [1% tryptone (w/v), 0.5% yeast extract (w/v), 1% NaCl (w/v) and 1.5% agar (w/v)] and grown in LB broth [1% tryptone (w/v), 0.5% yeast extract (w/v) and 1% NaCl (w/v)] at 37 °C with shaking (200 r.p.m.) unless otherwise stated.

Minimal salts glycerol glutamate (MSgg) medium (pH 7) contained 5 mM K_2_HPO_4_, 5 mM KH_2_PO_4_ and 100 mM MOPS pH 7, supplemented immediately before use with 0.5% (w/v) glutamic acid, 0.5% (w/v) glycerol, thiamine (2 µM) and a metal mix (2 mM MgCl_2_, 700 µM CaCl_2_, 50 µM MnCl_2_, 50 µM FeCl_3_ and 1 µM ZnCl_2_). For MSgg agar plates, 1.5% (w/v) agar was included in the base prior to autoclaving.

### Reagents and solutions

BugBuster® Master Mix (Merck Millipore Novagen, 71456-4) was used for biomass disruption and extraction of total protein to enable determination of PGA per unit protein in the biofilm. Where indicated, c*O*mplete™ EDTA-free protease inhibitor cocktail (Roche, 11836170001) was added. Protein concentrations were determined using the Pierce™ bicinchoninic acid (BCA) Protein Assay Kit (Thermo Scientific, 23227). Proteinase K (Qiagen, 19133) and DNase I (Qiagen, EN0521; 1 U µl^−1^) were used for the removal of protein and DNA, respectively. Methylene blue staining/assay solutions were prepared as 0.5% (w/v) methylene blue in 3% (v/v) acetic acid for gels and 25 µM methylene blue in water for microplate assays. Sulfuric acid (Sigma-Aldrich, 339741) and absolute ethanol (Sigma-Aldrich, 32221-2.5LM) were used during PGA precipitation.

SDS-PAGE was performed using standard Tris–glycine running buffer (25 mM Tris, 0.192 M glycine, 0.1% SDS and pH 8.3) and 4× SDS loading dye [60 mM Tris-HCl pH 8.8, 4% *β*-mercaptoethanol, 2% SDS (w/v), 10% glycerol (w/v) and 0.04% bromophenol blue (w/v)]. Coomassie staining used InstantBlue® (Abcam, ab119211). Precision Plus Protein™ Dual Colour Standards (Bio-Rad, 161–0394) were used as molecular weight markers.

### Purification of PGA for preparation of standards

PGA was purified from liquid cultures of *B. subtilis* NCIB 3610 Δ*tasA*. Single colonies were isolated by streaking onto LB agar and incubating at 37 °C for 16–24 h. A starter culture was prepared by inoculating one colony into 3 ml LB in a 50 ml tube and incubating at 37 °C with shaking (200 r.p.m.) for 3–4 h until OD_600_ reached 0.8–1.0. OD_600_ was measured in semi-micro cuvettes using a UV spectrophotometer after 1:10 dilution in sterile medium.

Four 250 ml baffled flasks containing 50 ml pre-warmed MSgg were inoculated to a starting OD_600_ of 0.025 and incubated at 50 °C with shaking (200 r.p.m.) for 24 h. Cultures were centrifuged at 4,000 r.p.m. for 10 min, and supernatants were pooled in a 1 l bottle without disturbing pellets. PGA standards were prepared from clarified culture supernatants rather than colony biofilm extracts to minimize co-extraction of cellular and matrix-associated components. The pooled spent supernatant (~200 ml) was adjusted to pH 2 using concentrated sulfuric acid, monitored using pH strips and incubated at 4 °C for 17–24 h.

Ice-cold 100% ethanol (200–800 ml) was added to precipitate PGA, followed by incubation at −20 °C for ≥24 h. Precipitated material was collected by centrifugation at 4,000 r.p.m. for 10 min in 50 ml tubes, repeating until all precipitate was recovered. Pellets were resuspended in a total of 10 ml 10 mM Tris-HCl pH 8.0 and lyophilized in pre-weighed tubes. PGA yield was determined by dry mass, and lyophilized PGA was stored at −20 °C with desiccant until use.

### Colony biofilm growth and biomass harvesting

Colony biofilms were grown on MSgg agar. Fresh liquid cultures were incubated for 4–6 h (OD_600_ >1), adjusted to OD_600_=1.0 in 500 µl Luria Broth (LB), and 5 µl was spotted onto MSgg agar plates and allowed to dry. Plates were incubated under the required experimental conditions, typically for 24 h. NCIB 3610 Δ*capB* was included as a PGA-negative control where indicated.

For harvest, 500 µl BugBuster® (± protease inhibitor) was aliquoted into labelled 1.5 ml tubes. Biofilm material was collected using a sterile 10 µl loop and transferred into BugBuster®. Samples could be stored at −20 °C overnight prior to processing. Biomass was disrupted by repeated passage through a 23G needle attached to a 1–3 ml syringe until viscosity decreased as far as possible. Samples were then sonicated (Q500 sonicator) at 30% amplitude using a 10-s timer with 1-s pulse on and 3-s pulse off, keeping samples on ice and cleaning the probe between samples.

Disrupted samples were incubated at 21–23 °C with agitation (300 r.p.m.) for 20 min and then centrifuged at 4 °C at 17,000 ***g*** for 10 min. Supernatants containing soluble proteins and PGA were transferred to fresh tubes and stored at −20 °C.

### Protein quantification

Protein concentrations of biofilm extracts were measured using the Pierce™ BCA assay according to the manufacturer’s instructions, using BugBuster®-compatible conditions. Microplates were incubated at 37 °C for 30 min, and absorbance was measured at 562 nm. Blank-corrected absorbance values were used to generate a protein standard curve, and the resulting regression equation was used to convert sample absorbance to protein concentration (µg ml^−1^). The values were used to adjust the biofilm extract samples to contain the same amount of protein prior to either SDS-PAGE analysis or PGA quantification.

### SDS-PAGE analysis and staining for PGA

To visualize PGA and extracted proteins, SDS-PAGE was performed using hand-cast gels consisting of 12% resolving gel and 7% stacking gel (cast at a 3:1 resolving:stacking ratio). Biofilm extracts were adjusted to contain 5–10 µg protein per lane (final volume 25 µl) using BugBuster® and mixed with SDS loading dye. Samples were heated at ~99 °C for 5 min, cooled and briefly centrifuged. Samples (25 µl) and protein ladder (5 µl) were loaded and electrophoresed at 180 V for ~1 h in Tris–glycine SDS running buffer until the dye front reached the bottom.

Gels were rinsed in water and stained with InstantBlue® Coomassie stain for 1 h with gentle rocking (≈50 r.p.m.), rinsed with water and then stained with 0.5% (w/v) methylene blue in 3% (v/v) acetic acid for 10–20 min with gentle rocking. Gels were washed in water until background staining was removed, and bands were clearly visible and then imaged using a ChemiDoc system.

### Microplate-based quantification of PGA using methylene blue

#### Preparation of PGA standards

Lyophilized PGA was weighed (10 mg) and dissolved in 1 ml BugBuster® to generate a 10 mg ml^−1^ stock, mixing thoroughly by vortexing. Working standards were prepared by serial dilution in BugBuster® (Table 1), vortexing between each dilution. Standards were stored at 4 °C and vortexed before use. Standards above 2 mg ml^−1^ were not used for quantification due to non-linearity observed at higher concentrations.

**Table 1.** Preparation of PGA standards

PGA conc. (mg ml^−1^)	Stock (mg/ml)	Vol. of stock required (µl)	Vol. of BugBuster® required (µl)	Total vol. before use (µl)
5	10	500	500	1,000
4	10	400	600	1,000
3	5	480	320	800
2	4	400	400	800
1	10	80	720	800
0.75	2	300	500	800
0.5	1	400	400	800
0.25	2	100	700	800
0.125	1	100	700	800
0.025	0.5	40	760	800

#### Sample preparation and enzymatic treatment

Biofilm extracts were adjusted to 10 µg protein per 25 µl (i.e. 20 µg protein in 50 µl; 0.4 µg µl^−1^) using BugBuster® as diluent. Where used, DNase I treatment was performed prior to proteolysis. Samples were treated with Proteinase K (1 µl per sample) and incubated at 56 °C for ≥90 min (up to 4 h), followed by a brief pulse centrifugation. Treated samples were diluted to give final inputs equivalent to 1 µg and 0.1 µg protein per 25 µl. An additional 1:10 dilutions were prepared as needed to ensure sample signals fell within the linear range of the PGA standard curve.

#### Assay setup and absorbance measurements

For each well, 25 µl of PGA standard (in duplicate) or prepared sample was added to a 96-well microplate, followed by 275 µl of 25 µM methylene blue in water (final volume 300 µl). Plates were mixed gently to avoid bubbles and incubated at room temperature (~23 °C) for 10 min. Absorbance spectra were measured from 400 to 800 nm (2 nm increments) using a PHERAstar® FSX plate reader, or absorbance was recorded at 564 and 664 nm.

#### Data processing and calculation of PGA concentration

Raw absorbance values were background-corrected by subtracting the blank well reading from standards and samples. Standard curves were generated independently at 564 and 664 nm using known PGA concentrations, and linear regression equations (with *R*^2^ reporting) were used to convert sample absorbance to PGA concentration. PGA estimates derived from 564 and 664 nm were averaged, and final values were expressed as mg PGA per µg protein (and convertible to mg PGA per mg protein as required). Data analysis was performed in Microsoft Excel.

## Results

A literature search revealed that the spectral profile (400–800 nm) of methylene blue changes when bound to PGA [13]. On that basis, a quantification assay for samples isolated from biofilms was developed.

### Optimization of the strain used to purify PGA as a standard

**Fig. 1.**
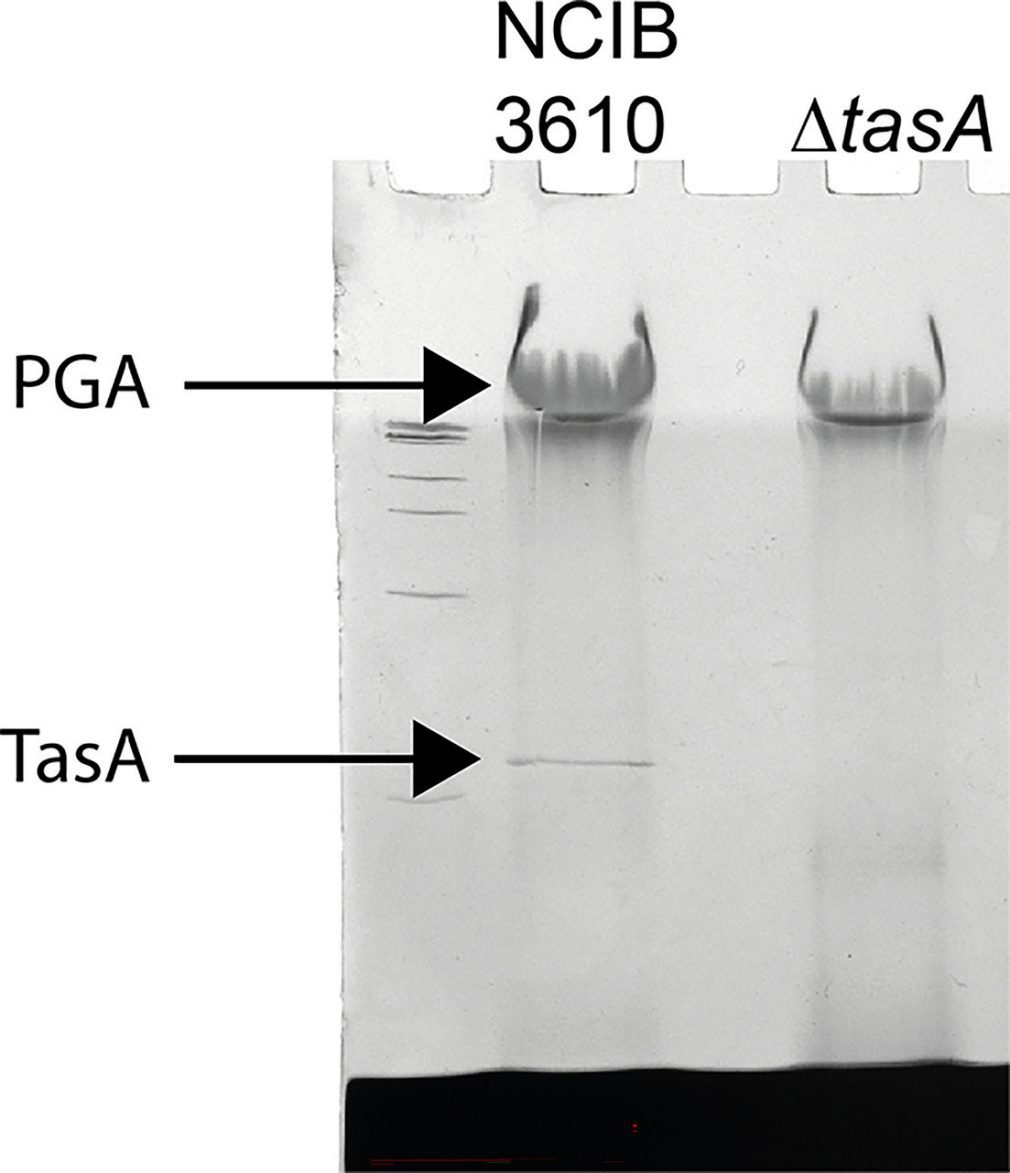
Visualization of PGA by SDS-PAGE. Acrylamide gel stained with Coomassie blue and methylene blue dye showing the presence (or absence) of TasA from purified PGA derived from NCIB 3610 or NCIB 3610 Δ*tasA* extracts. Gel stained with Coomassie blue dye rinsed and re-stained with methylene blue before imaging on a Chemidoc imaging system. The arrow indicates TasA and PGA bands.

The first step required to develop the quantification assay was the generation of a high-quality sample of PGA to allow preparation of an accurate standard curve. We chose to purify PGA from liquid cultures of a *B. subtilis* NCIB 3610 Δ*tasA* strain incubated at 50 °C. This enabled the preparation of standards of known concentration that lacked a major protease-resistant extracellular protein (Fig. 1) [14]. The temperature selection (of 50 °C) was made based on previous findings as it was shown to increase PGA production [4]. The band labelled PGA is absent when the PGA biosynthetic gene cluster is disrupted [5]. After isolation, the sample of PGA was freeze dried for long-term storage.

### PGA does not interfere with protein measurements

**Fig. 2.**
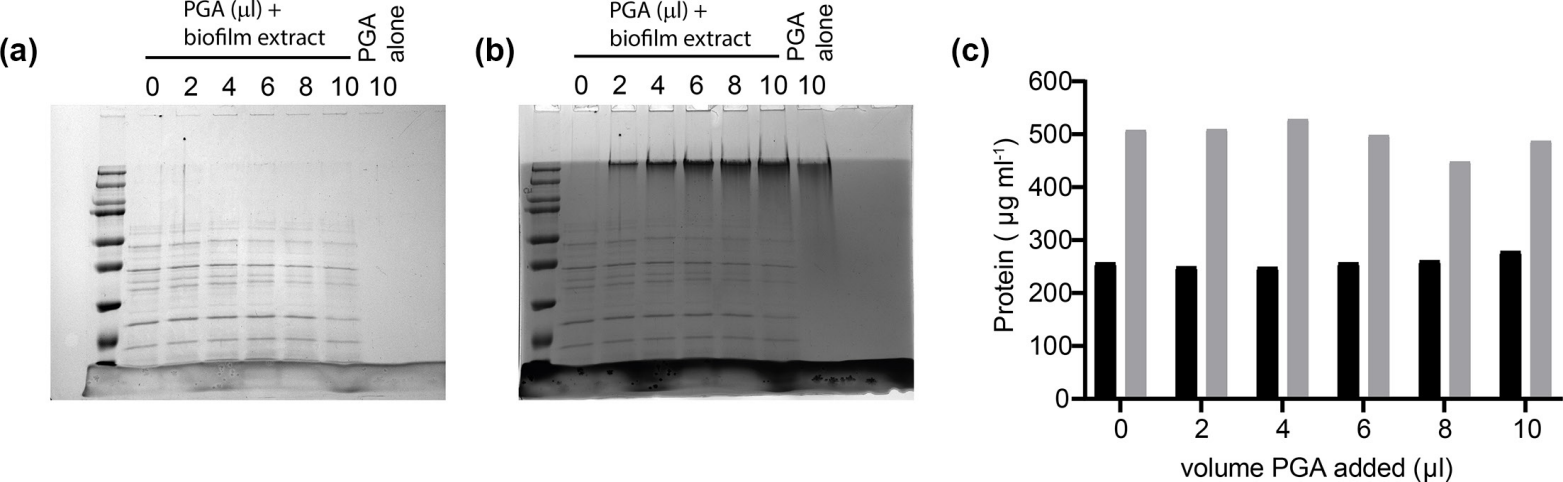
PGA does not interfere with protein measurements. (**a, b**) Acrylamide gels loaded with biofilm extracts where each contained 7.5 µg protein and increasing volumes of PGA (0, 2, 4, 6, 8 and 10 µl PGA) stained with Coomassie blue (**a**) and Coomassie blue and then methylene blue (**b**). (**c**) Quantification of known concentrations of protein (*n*=2) in samples in the presence of increasing PGA additions.

Accurate adjustments of biofilm samples to equivalent protein amounts require reliable measurement of protein concentrations in biofilm extracts. To determine whether PGA interferes with protein quantification, aliquots of a single biofilm extract were supplemented with increasing volumes of purified PGA while maintaining the same starting biofilm extract and therefore the same protein content (Fig. 2a, b). Protein concentrations were then re-measured using the BCA assay. No substantial differences in measured protein concentrations were observed across samples containing increasing amounts of added PGA (Fig. 2c), indicating that PGA does not interfere detectably with protein quantification under the conditions used.

### Removing interference in measurements from other polymers in biofilm extracts

**Fig. 3.**
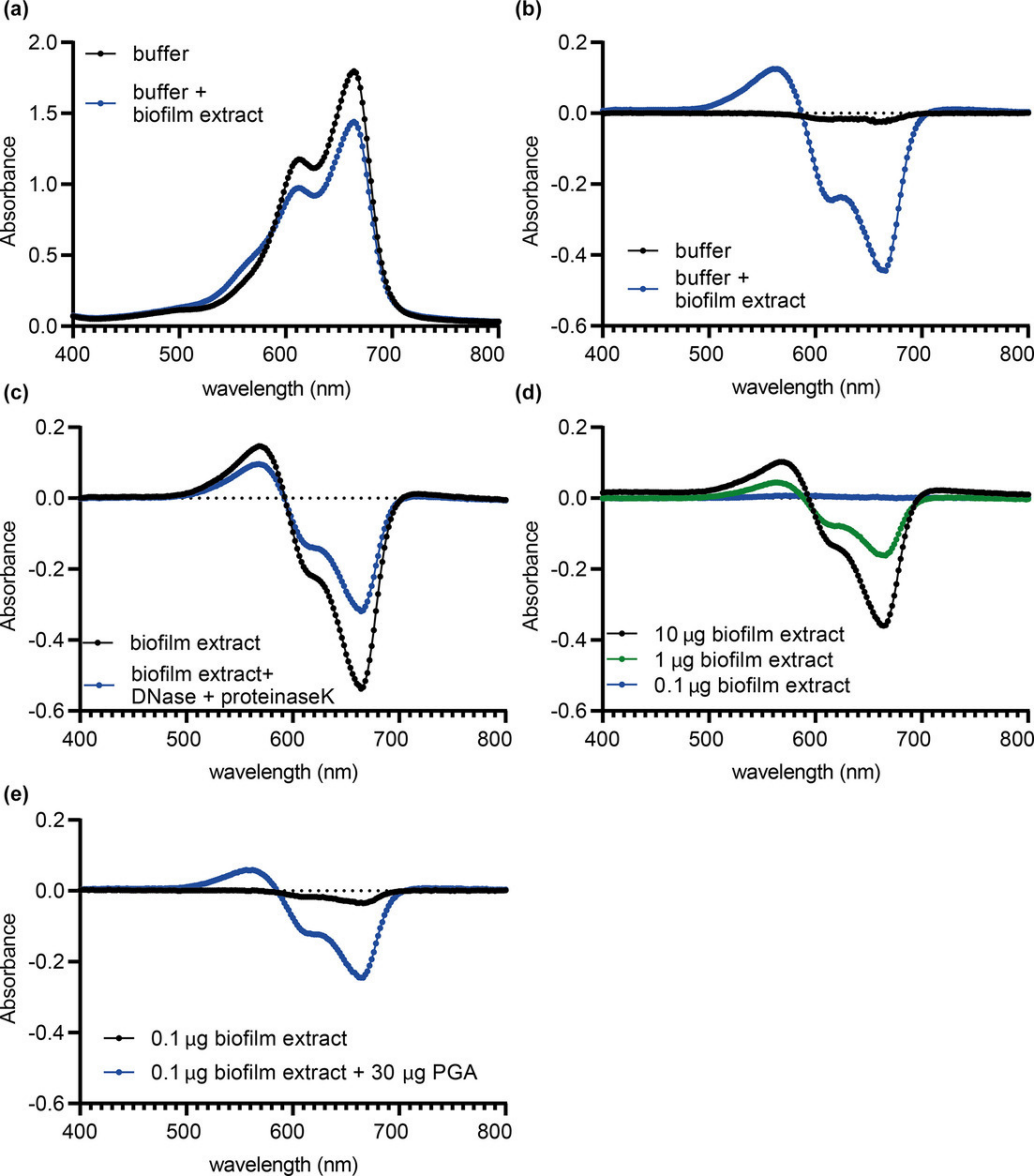
Protease and DNase treatment reduces background interference from biofilm extracts. Spectral profiles (400–800 nm) of methylene blue incubated with BugBuster® or biofilm extracts from the PGA-negative strain NCIB 3610 Δ*capB*. Unless otherwise indicated, each well contained 275 µl methylene blue solution and 25 µl BugBuster® or biofilm extract. (a) Raw spectral profiles of methylene blue mixed with BugBuster® alone (black dotted line) or untreated biofilm extract from NCIB 3610 Δ*capB* (blue dotted line). Untreated biofilm extracts produced pronounced changes in the methylene blue spectrum, particularly near 564 and 664 nm. (b) Background-corrected spectral profiles corresponding to panel (a), obtained by subtracting the methylene blue-only profile from each sample. Biofilm extracts from the PGA-negative strain produced PGA-like spectral changes, indicating interference from non-PGA components. (c) Spectral profiles of methylene blue incubated with untreated biofilm extract (black dotted line) or biofilm extract treated with proteinase K and DNase (blue dotted line). Enzymatic treatment reduced interference from proteins and extracellular DNA but did not eliminate the background signal completely. (d) Spectral profiles of methylene blue incubated with treated NCIB 3610 Δ*capB* biofilm extracts diluted to contain 10 µg (black dotted line), 1 µg (green dotted line) or 0.1 µg (blue dotted line) protein equivalent per well. Progressive dilution reduced the magnitude of background interference. (e) Background-corrected spectral profiles of methylene blue incubated with treated and diluted NCIB 3610 Δ*capB* biofilm extract (0.1 µg protein equivalent; black dotted line) or the same extract supplemented with 30 µg purified PGA (blue dotted line), demonstrating detectable PGA-dependent spectral changes above the residual background signal.

The compatibility of biofilm extracts with the methylene blue assay was next assessed. Methylene blue was incubated with BugBuster® reagent or water, and spectral profiles were compared with those obtained using untreated biofilm extracts (Fig. 3a, b). BugBuster® and water controls showed minimal deviation from the methylene blue-only profile. In contrast, biofilm extracts from the PGA-negative strain NCIB 3610 Δ*capB* produced pronounced alterations in the methylene blue spectrum, including increased absorbance near 564 nm and decreased absorbance near 664 nm, similar to the spectral changes observed with purified PGA. These results indicate that untreated biofilm extracts contain components that interfere with the assay and generate PGA-like spectral profiles even in the absence of PGA. Biofilm extracts from the Δ*capB* strain were therefore used to define the background interference signal.

To reduce this interference, biofilm extracts were treated with proteinase K and DNase to remove proteins and extracellular DNA, respectively. These treatments reduced the magnitude of the interfering signal but did not eliminate it completely (Fig. 3c), indicating that additional biofilm components contribute to the observed spectral changes.

As interference appeared proportional to the amount of biofilm extract present, samples were diluted to lower protein-equivalent inputs. Progressive dilution of Δ*capB* biofilm extracts produced corresponding reductions in absorbance changes across the spectrum (Fig. 3d), demonstrating that background interference could be reduced to low levels by limiting the amount of extract used in the assay.

Finally, purified PGA was added to treated and diluted Δ*capB* biofilm extracts. Under these conditions, the expected PGA-dependent spectral shift was clearly detectable relative to the residual background signal (Fig. 3e). Together, these results demonstrate that methylene blue binding is not intrinsically specific for PGA in untreated biofilm extracts but that a combination of enzymatic treatment and sample dilution reduces background interference sufficiently to allow reproducible detection and quantification of PGA in biofilm-derived samples.

### Establishment of a robust PGA standard curve

**Fig. 4.**
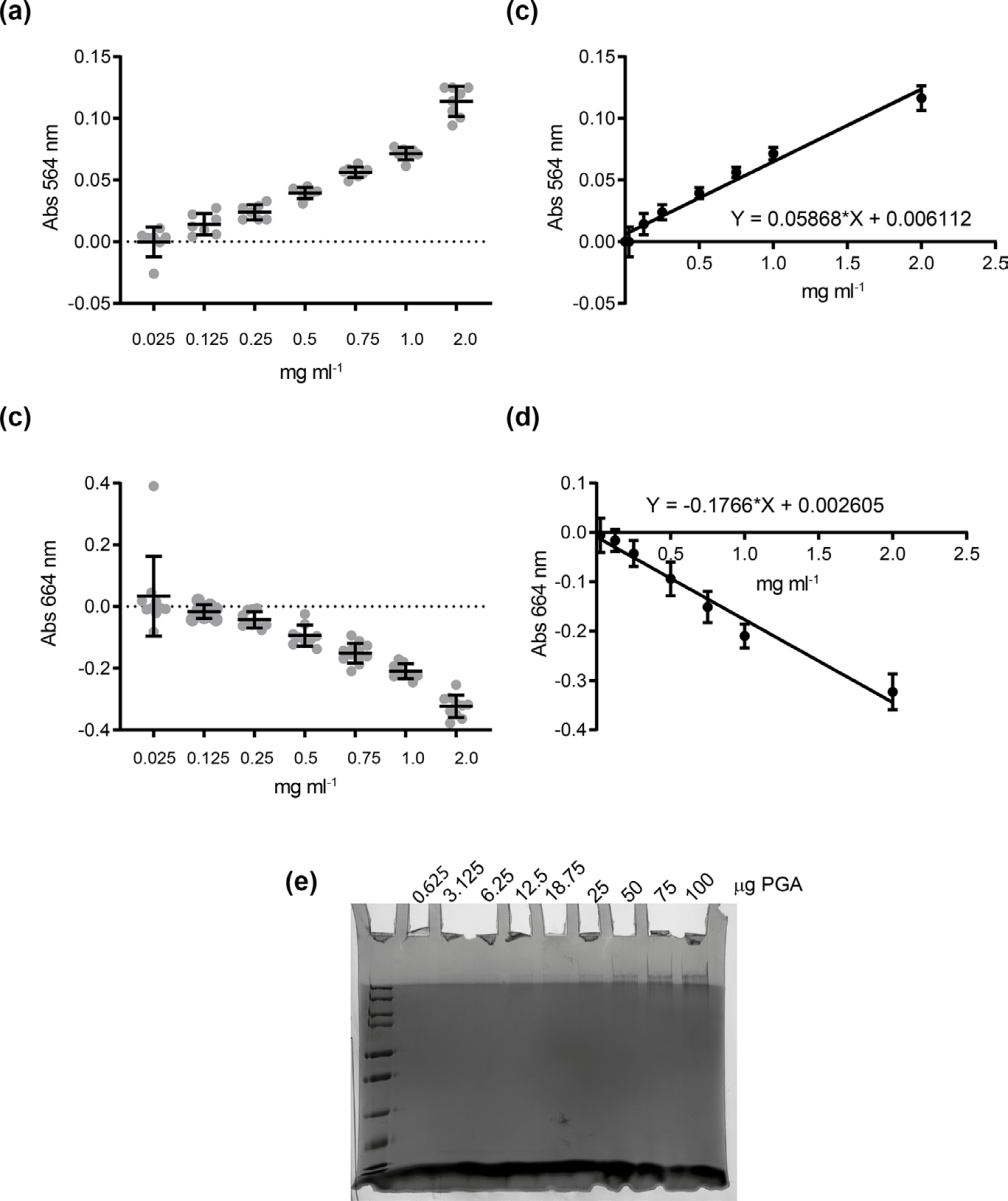
PGA standard curve. (**a**) Replicates of PGA standards (0.025−2 mg ml^−1^, *n*=7) with absorbance readings at 564 nm (after background correction). Readings plotted on the same graph to show the spread of data for each standard. (**b**) Equation of the line for averaged PGA standards (0.25−2 mg ml^−1^, *n*=7), readings at 564 nm. Example of the equation of the line used for quantification of samples with unknown PGA content. (**c**) Replicates of PGA standards (0.25−2 mg ml^−1^, *n*=9) with absorbance readings at 664 nm (after background correction). Readings plotted on the same graph to show the spread of data for each standard. (**d**) Equation of the line for averaged PGA standards (0.25−2 mg ml^−1^, *n*=9), readings at 664 nm. Example of the equation of the line used for quantification of samples with unknown PGA content. Note: Sample readings for both 564 and 664 nm were used with respective equations to quantify PGA, and quantities were then averaged for a more accurate PGA value which was converted to mg PGA/µg protein. (**e**) PGA becomes visible for samples containing >10 µg.

Following optimization of sample preparation, the linear range of the quantification assay was determined using standard curves prepared from independent PGA purifications. Replicates clustered tightly across batches, indicating strong reproducibility (Fig. 4a, c). An upper limit of 2 mg ml^−1^ PGA was identified as the linear range for the assay, as higher concentrations deviated from linearity (Fig. 4).

Final standard concentrations ranged from 0.025 to 2 mg ml^−1^ (Table 1). Absorbance readings at both 564 and 664 nm were used to calculate PGA concentrations, with values averaged to improve accuracy. When compared to SDS-PAGE-based detection, the methylene blue microplate assay demonstrated superior sensitivity, detecting ~3 µg PGA in 25 µl, whereas SDS-PAGE required ≥10 µg for reliable detection (Fig. 4e).

## Discussion

In this study, we developed and validated a methylene blue–based assay for quantifying PGA in biofilm-derived samples. Building on previous observations that methylene blue exhibits a distinct spectral shift upon binding PGA [13], we adapted this approach for complex biological samples, where interference from other extracellular biofilm matrix components can present a significant challenge. A key requirement for assay development was the generation of a high-purity PGA standard. Use of the NCIB 3610 Δ*tasA* strain effectively eliminated contamination from TasA, a major extracellular matrix protein known to resist proteolysis and complicate downstream analyses [14]. Growth at elevated temperature further enhanced PGA yield, consistent with previous findings [4], enabling the preparation of reproducible standard curves for the quantification of various samples.

Accurate adjustment of PGA content to protein levels in a sample is essential for comparing material recovered from biofilms formed by different strains or growth conditions. Our results demonstrate that PGA does not interfere with protein quantification, supporting the use of protein-normalized PGA values. However, it should be noted that the BCA assay used for protein determination is based on the reduction of Cu²^+^ ions [15] and can therefore respond to reducing compounds in addition to proteins, including reducing sugars and degradation products of extracellular polysaccharides that may be present in biofilm extracts [16]. In principle, such interference could be reduced by enzymatic removal of extracellular polysaccharides prior to protein quantification; however, suitable enzyme treatments were not available. Alternatively, genetic approaches targeting exopolysaccharide production would be expected to alter biofilm composition and physiology in ways that could influence PGA production independently of the quantification method [17]. We therefore adopted a pragmatic approach in which protein normalization was retained while recognizing that extracellular polysaccharides could make a minor contribution to the measured signal. We found that untreated biofilm extracts substantially altered methylene blue spectral profiles, even in the absence of PGA, highlighting the presence of confounding components. The combined use of proteinase K, DNase and sample dilution substantially reduced background interference and allowed PGA-dependent spectral changes to be distinguished from residual signal. While these treatments do not completely eliminate interference from other biofilm components, they provide conditions under which PGA can be quantified reproducibly in complex biofilm extracts. The resulting PGA standard curve was robust across independent PGA preparations and exhibited a well-defined linear range up to 2 mg ml^−1^. Importantly, the methylene blue microplate assay showed greater sensitivity than SDS-PAGE-based detection, making it better suited for quantification of low-abundance PGA in biofilm samples. While SDS-PAGE remains useful for visual confirmation, the microplate assay provides a faster, more sensitive and quantitative alternative.

Overall, the assay provides a practical and reliable method for quantifying PGA in biofilm samples after appropriate sample treatment. Although some background interference from biofilm-derived components remains, the assay is well suited as a rapid screening method for comparative analysis of PGA production across multiple strains or growth conditions. The microplate-based format allows large numbers of samples to be analysed efficiently with minimal sample volumes, enabling identification of candidate high-yield PGA producers. Samples identified as promising using this approach could subsequently be analysed using more specific analytical methods, such as chromatographic techniques, to confirm PGA yields. This two-stage strategy provides a practical balance between analytical specificity and experimental throughput.

## Supplementary material

10.1099/acmi.0.001162.v4 Supplementary Material 1.

## Abbreviations

BCA: bicinchoninic acid
LB: Luria Broth
MSgg: minimal salts glycerol glutamate
PGA: poly-*γ*-glutamic acid

## References

[1] Shih I-L, Van Y-T (2001). The production of poly-(γ-glutamic acid) from microorganisms and its various applications. Bioresource Technol.

[2] Shih I-L, Van Y-T, Shen M-H (2004). Biomedical applications of chemically and microbiologically synthesized poly(glutamic acid) and poly(lysine). Mini Rev Med Chem.

[3] Hsueh Y-H, Cozy LM, Sham L-T, Calvo RA, Gutu AD (2011). DegU-phosphate activates expression of the anti-sigma factor FlgM in *Bacillus subtilis*. Mol Microbiol.

[4] Morris RJ, Stevenson D, Sukhodub T, Stanley-Wall NR, MacPhee CE (2022). Density and temperature controlled fluid extraction in a bacterial biofilm is determined by poly-γ-glutamic acid production. NPJ Biofilms Microbiomes.

[5] Stanley NR, Lazazzera BA (2005). Defining the genetic differences between wild and domestic strains of *Bacillus subtilis* that affect poly-gamma-dl-glutamic acid production and biofilm formation. Mol Microbiol.

[6] Yu Y, Yan F, Chen Y, Jin C, Guo J-H (2016). Poly-γ-glutamic acids contribute to biofilm formation and plant root colonization in selected environmental isolates of *Bacillus subtilis*. Front Microbiol.

[7] Schallmey M, Singh A, Ward OP (2004). Developments in the use of *Bacillus* species for industrial production. Can J Microbiol.

[8] Pedreira T, Elfmann C, Stülke J (2022). The current state of SubtiWiki, the database for the model organism *Bacillus subtilis*. Nucleic Acids Res.

[9] Ashiuchi M, Misono H (2002). Biochemistry and molecular genetics of poly-gamma-glutamate synthesis. Appl Microbiol Biotechnol.

[10] Bamford NC, MacPhee CE, Stanley-Wall NR (2023). Microbial Primer: an introduction to biofilms – what they are, why they form and their impact on built and natural environments. *Microbiology*.

[11] Arnaouteli S, Bamford NC, Stanley-Wall NR, Kovács ÁT (2021). Bacillus subtilis biofilm formation and social interactions. Nat Rev Microbiol.

[12] Kalamara M, Abbott JC, MacPhee CE, Stanley-Wall NR (2021). Biofilm hydrophobicity in environmental isolates of *Bacillus subtilis*. *Microbiology*.

[13] Chatterjee PM, Datta S, Tiwari DP, Raval R, Dubey AK (2018). Selection of an effective indicator for rapid detection of microorganisms producing γ-Polyglutamic Acid and its biosynthesis under submerged fermentation conditions using *Bacillus methylotrophicus*. Appl Biochem Biotechnol.

[14] Erskine E, Morris RJ, Schor M, Earl C, Gillespie RMC (2018). Formation of functional, non‐amyloidogenic fibres by recombinant *Bacillus subtilis* TasA. Mol Microbiol.

[15] Walker JM (1994). The bicinchoninic acid (BCA) assay for protein quantitation. Methods Mol Biol.

[16] Hussain H, Ngaini Z, Chong NFM (2018). Modified bicinchoninic acid assay for accurate determination of variable length reducing sugars in carbohydrates. Int Food Res J.

[17] Porter M, Davidson FA, MacPhee CE, Stanley-Wall NR (2022). Systematic microscopical analysis reveals obligate synergy between extracellular matrix components during *Bacillus subtilis* colony biofilm development. *Biofilm*.

