# Supplementary material for: Microplate-based quantification of poly-γ-glutamic acid levels in biofilm samples
Source: Access Microbiol. 2026 Jun 9;8(6):001162.v4. doi: 10.1099/acmi.0.001162.v4 (PMC13249132; doi:10.1099/acmi.0.001162.v4)
Supplement: Supplementary Material 1. [file acmi-8-01162-s001.pdf]

Supplemental Material

Microplate-Based Quantification of Poly-γ-Glutamic Acid (γ-PGA) levels in Biofilm Samples

David Stevenson [1], Cait E MacPhee [2], Nicola R Stanley-Wall [1]

[1]Division of Molecular Microbiology, School of Life Sciences, University of Dundee, Dundee, DD1 5EH UK

[2]National Biofilms Innovation Centre, School of Physics and Astronomy, The University of Edinburgh, Edinburgh, EH9 3FD UK

Corresponding author. Nicola Stanley-Wall n.r.stanleywall@dundee.ac.uk

David Stevenson - https://orcid.org/0000-0003-1812-2658

Cait MacPhee - https://orcid.org/0000-0001-8313-8387

Nicola Stanley-Wall - https://orcid.org/0000-0002-5936-9721

Contents

Supplemental Material 1
Microplate-Based Quantification of Poly-γ-Glutamic Acid (γ-PGA) levels in Biofilm Samples . 1
Biological materials 3
Reagents 3
Solutions 3
Recipes 4
Laboratory Supplies 6
Equipment 6
Software and databases 7
Procedure 8
Purify PGA for use in the standard curve 8
Grow biofilms and harvest 9
Analysis of PGA by electrophoresis 11
Quantification of PGA 12
Part 1: Prepare PGA standard curve 12
Part 2: Prepare the samples 12
Part 3 – Set up the PGA assay plate 13
Part 4: Calculate the PGA concentration in the sample 13

Biological materials

1. *Bacillus subtilis* NCIB 3610 Δ*tasA* (used to prepare PGA standards – see validation section)
2. *Bacillus subtilis* NCIB 3610 (wild type)
3. *Bacillus subtilis* NCIB 3610 Δ*capB* (negative control)

Reagents

1. Ethanol absolute ≥99.8% (Sigma-Aldrich®, catalogue number: 32221-2.5LM)
2. Bugbuster® Master Mix (Merck Millipore® Novagen®, catalogue number: 71456-4, storage temperature 2-8°C)
3. cOmplete™ EDTA-free Protease Inhibitor Cocktail (Roche, catalogue number: 11836170001, storage 2-8°C. Stock solution is stable for 1-2 weeks at 2 to 8 °C, or at least 12 weeks at -15 to -25°C)
4. Pierce™ BCA Protein assay kit (Thermo Scientific™, catalogue number: 23227, store at room temperature)
5. Qiagen® Proteinase K >600 mAU/ml (Qiagen®, catalogue number: 19133 storage temperature 15-25°C).
6. Qiagen® DNase1 (1U/µl) (Qiagen®, catalogue number: EN0521)
7. Sulfuric acid (Merck, Sigma-Aldrich®, catalogue number: 339741-100 ml)
8. InstantBlue® Coomassie protein stain (Abcam, catalogue number: ab119211)
9. Precision-Plus Protein™ Dual Colour Standards Protein ladder (Bio-Rad, catalogue number: 161-0394)
10. ProtoGel 30% (w/v) 37.5:1 Acrylamide to Bisacrylamide Stabilized Solution (National Diagnostics, catalogue number: EC-890, store at room temperature in dark area)
11. TEMED (VWR, catalogue number: 443083G)

Solutions

1. Tris-HCl 10 mM
2. NaCl 0.14 mM
3. Methylene blue dye (see recipes)
4. 4Xx SDS-PAGE loading dye (see recipes)
5. 1XSDS buffer (see recipes)

Recipes

1. LB agar plate (autoclaved at 121°C prior to pouring, 25 ml per plate, storage conditions 2-8°C for 2-4 weeks)
    - 1% (w/v) tryptone
    - 0.5% (w/v) yeast extract
    - 1% (w/v) NaCl
    - select agar 1.5% (w/v)
2. LB broth (autoclaved at 121°C, storage room temperature for several months)
    - 1% (w/v) tryptone
    - 0.5% (w/v) yeast extract
    - 1% (w/v) NaCl
3. Minimal Salts glycerol glutamate (MSgg) media (final concentrations)

    MSgg base pH 7 (autoclaved, storage room temperature for several months):
    - 5 mM di-potassium phosphate
    - 5 mM di-hydrogen phosphate
    - 100 mM 3-(N-morpholino) propanesulfonic acid pH 7 (MOPS)

    The base is supplemented with "metal mix" and glutamic acid immediately before use:

    0.5% glutamic acid (w/v) (10% glutamic acid (w/v) stock autoclaved at 121°C, storage room temperature, months).

    "metal mix" (filter sterilised through a 0.22 µm MF-Millipore™ MCE membrane filter, storage 2-8°C):
    - 2 mM $MgCl_2$
    - 700 µM $CaCl_2$
    - 50 µM $MnCl_2$
    - 50 µM $FeCl_3$
    - 1 µM $ZnCl_2$
    - 2 µM thiamine
    - 0.5% glycerol (w/v)

    For agar plates include 1.5 % agar final (w/v) in the media base at the point of autoclaving.
4. SDS Running Buffer (storage room temperature, months)
    - 25 mM Tris
    - 0.192 M glycine

- 0.1% SDS
- pH 8.3

5. SDS loading dye (storage room temperature, months)
    - 60mM Tris-HCI pH8.8
    - 4% (v/v) β-Mercaptoethanol
    - 2% SDS (w/v)
    - 10% Glycerol (v/v)
    - 0.04% (w/v) Bromophenol blue
6. Methylene blue dye (storage room temperature, months)
    - 0.5% methylene blue (w/v)
    - 3% Acetic acid (v/v)
7. lower buffer (for acrylamide gels) storage 2-8°C
    - 1.5 M Tris-HCl pH 8.8
8. Upper buffer (for acrylamide gels) storage 2-8°C
    - 0.5 M Tris-HCL pH 6.8
9. Resolving gel (Component and required volume for 2 gels)
    - Lower Buffer 2.5 ml
    - 10% Sodium dodecyl sulphate (SDS) 100 µl
    - 10% Ammonium Persulfate (APS) 100 µl
    - TEMED 10 µl
    - 30% (w/v) Protogel (37.5 :1 acrylamide to bisacrylamide Stabilized Solution) 4 ml
    - H_2O 3.29 ml
10. Stacking gel
    - Component Volume for 2 gels
    - Upper gel buffer 1.25 ml
    - 10% Sodium dodecyl sulphate (SDS) 50 µl
    - TEMED (add last) 5 µl
    - 10% Ammonium Persulfate (APS) 50 µl
    - 30% (w/v) Protogel (37.5 :1 acrylamide to bisacrylamide Stabilized Solution) 1 ml
    - H_2O 2.73 ml

Laboratory Supplies

1. Reaction microtube 1.5 ml (Sarstedt®, catalogue number: 72.690.001)
2. Brand® microcentrifuge tube 2 ml (Brand®, catalogue number: BR780546-500EA)
3. Greiner 96 well plates (Greiner Bio-one, catalogue number: 655101)
4. Greiner 50 ml Falcon® CellStar® tube (Greiner Bio-one, catalogue 227261)
5. pH strips Fix 0-14 (Fisher brand™, catalogue number 10642751)
6. Agani™ hypodermic needles 23 G X 1$^{1/4}$ (Terumo® catalogue number: AN*2332R1)
7. Syringe 1 ml (Fisher scientific Terumo®, catalogue number: MDSS01SE)
8. Syringe 3 ml (Fisher scientific Terumo®, catalogue number: MDSS03SE)
9. Semi-micro cuvettes 3 ml (Sarstedt®, catalogue number: 67.742)
10. Sterile reagent reservoir (VWR®, catalogue number: 613-1174)
11. 5 ml Serological pipettes (Sarstedt®, catalogue number: 86.1253.001)
12. 10 ml Serological pipettes (Sarstedt®, catalogue number: 86.1254.001)
13. 25 mL Serological pipettes (Sarstedt®, catalogue number: 86.1685.001)
14. 10 µL pipette tips (Starlab catalogue number: S1111-3000)
15. 20 µL pipette tips, ART™ barrier (Thermo Fisher Scientific, catalogue number: 2149P-05-HR)
16. 200 µL pipette tips (Starlab catalogue number: S1111-1006)
17. 1,250 µL pipette tips (Starlab catalogue number: S1112-1020)
18. 10 µl Loops (VWR®, catalogue number: 612-9359)
19. 30 ml universal container (Greiner Bio one, catalogue number: 201150)
20. 90 mm Petri dish (Thermo Scientific™, catalogue number: 101R20)
21. Membrane filter 0.22 µm (Merck, MF-Millipore™catalogue number: GSWP04700)

Equipment

1. Pipettes P10, P20, P200, P1000 (Gilson™, pipetman catalogue numbers: F144055M, F144056M F144058M, F144059M)
2. Pipetboy2 (integra, catalogue number: 155015)
3. 1L Duran® bottles
4. Vortex mixer (VWR international)
5. Laminar flow cabinet (Esco®, model: LVG-4AG-F8)
6. Shaking incubator or warm room (37°C)
7. Platform shaker (New Brunswick Scientific model: InnOva® 2100)
8. Spectrophotometer (Thermo electron corporation, model: GENESYS™ 10 UV-Vis)

9. 250 ml Erlenmeyer(conical) flasks (Schott, Duran® conical flask)
10. Water bath or incubator (50°C) Innova™ 3100 water bath shaker New Brunswick Scientific
11. Plate reader (BMG Labtech, model: PHERAstar® FSX plate reader)
12. Centrifuge (Falcon® tubes) (Sigma, model: sigma benchtop 3-16 KL)
13. -20°C Freezer (Biocold Laboratory Freezer) *note must be spark free
14. Centrifuge (microtube) (Thermo Electron Corporation, model: Heraeus Fresco™ 17 centrifuge)
15. Incubator (Thermo Scientific, Heratherm™ Incubator)
16. Sonicator® (Q sonica sonicators®, model: Q500, catalogue: Q500-110).
17. Thermomixer (Eppendorf, model: thermomixer comfort 5355900.012)
18. Freeze dryer (model: alpha 1-2 LD plus freeze dryer, part no. 101521, 101522, 101527)
19. Gel electrophoresis Kit (BioRad, PowerPac™ Basic, BioRad, Mini-Protean® Tetra Cell, BioRad, Mini Protean® 3 Cell)
20. Imager gel documentation and analysis system, ChemiDoc gel imaging system (VWR® Imager CHEMI premium, catalogue 730-1469)

Software and databases

1. Microplate reader software, MARS Data analysis software 3.01 R2 PHERAstar 4.00R4 Firmware 1.22 FSX V6.20 Edition2 V6.20 V1.33/V2.00*1 V5.02 R3 31.07.2024
2. Excel, Microsoft® Excel® for Microsoft 365 MSO (Version 2506 Build 16.0.18925.20076) 64-bit

Procedure

Purify PGA for use in the standard curve

1. Under sterile conditions, streak out the PGA producing strain (e.g. Δ*tasA* strain) on an LB agar plate for single colonies and incubate the plate at 37°C allowing the bacteria to grow for 16-24 hours. For the rationale for why the Δ*tasA* strain was used see validation section.
2. Under sterile conditions, prepare a liquid culture by inoculating a single colony from the agar plate into a 50 ml Falcon® tube containing 3 mL LB. Incubate at 37°C with shaking at 200 rpm typically for 3-4 hours until an OD_{600} of between 0.8-1.0 is reached (Measure OD).
3. Measure culture OD, under sterile conditions dilute sample 1 in 10 in a cuvette and measure and record the OD_{600} in spectrophotometer.
4. Under sterile conditions, prepare four 250 ml flasks each containing 50 ml MSgg liquid medium (should be prepared and preheated at 30°C during incubation of bacterial culture).
5. Using the final OD of the liquid culture (step-2), calculate the required culture volume for a starting OD of 0.025 (in 50 ml), remove that volume of MSgg from each flask and inoculate with the required volume of culture (if required, pellet cells, wash with MSgg and resuspend in MSgg prior to measuring OD and inoculation step).
6. Incubate the prepared flasks at 50°C with shaking at 200 rpm for 24 hours.
7. After incubation, transfer the cultures to 50 ml Falcon® tubes (use same tubes throughout centrifugation). Centrifuge cells at 4000 rpm for 10 minutes.
8. Transfer the supernatant (called spent culture supernatant) to a 1L Duran® bottle (be careful not to disturb the cell pellet).
9. Repeat steps 7 & 8 until all the culture supernatant has been collected.
10. Optional step (or skip to step 13): Suspend the cell material into 2.5 ml 0.14 mM NaCl
11. Centrifuge cells at 4000 rpm for 10 minutes to pellet.
12. Add supernatant to the spent culture media.
13. At this point you will have around 200 mL spent culture supernatant.
14. Adjust the pH to pH 2 using concentrated sulphuric acid. Regularly check the pH with pH strips (Fisherbrand®) as required.
15. Place the Duran® bottle at 4°C for 17-24 hours.
16. Add 200 (up to a max of 800) mL of 100% (v/v) ethanol (ice cold) and incubate at -20°C in a Duran® bottle for at least 24 hours to precipitate the PGA (if it suits this step can be several days).

17. Decant the contents of the Duran® bottle into two 50 mL Falcon® tubes and centrifuge at 4000 rpm for 10 minutes (use the same Falcon® tubes throughout to concentrate the PGA).
18. Remove and discard the supernatant. Repeat steps 17-18 until all the precipitate is collected in the 2 Falcon® tubes.
19. Remove as much of the liquid as possible.
20. Resuspend material in 10 ml 10 mM Tris-HCl pH 8.0 (total).
21. Follow local procedures or protocols (depending on equipment available) to freeze dry the collected PGA in pre-weighted tubes (e.g. Falcon® tubes with holes in lid).
22. Measure the dry weight of the purified PGA.
23. Transfer the PGA to a suitable container and seal tightly, store the material at -20°C until required with desiccant.

Grow biofilms and harvest

1. Prepare and grow biofilms for testing as required – use your own methods for this and ideally include a *capB* (or related) mutant that cannot produce PGA as a negative control.

   Example summary of our method:

- Colony biofilms were prepared from fresh liquid cultures grown for 4-6 hours until the required optical density (OD) was reached (>1).
- Cultures were removed from incubation and OD_{600} measured, cultures were then adjusted to the desired OD_{600} of 1.0 in 500 µl LB.
- 5 µl of the culture was spotted onto MSgg agar plates (Branda et al., 2001) as required and allowed to dry.
- Plates were prepared in duplicate with controls and incubated at the required conditions.
- Normally, biofilms were incubated for 24 hours before harvesting.

2. For each biofilm sample to be collected, aliquot 500 µL Bugbuster® (optional with protease inhibitor) into an Eppendorf tube ready for harvesting (tubes should be pre-prepared and labelled as necessary for identification. Bugbuster® Master Mix is used for lysing cells and protein extraction). NOTE: biofilms may grow to occupy large portions of the plate, biofilms with increased footprint measurements may require multiple tubes or larger tubes to collect all the biomass, (ensure tubes are not filled to the top to allow for sample expansion when frozen), adapt as necessary.

3. At the time point of interest, collect the biofilm material (prepared at step 1) from the agar plate using a 10 µL loop and place in the prepared Eppendorf tube(s), briefly mix. NOTE: At this point the nature of the biomass could be highly different – it could be dry and compact, or it could be highly mucoid and challenging to scoop up.
4. The samples can be stored at -20°C overnight. This is not a necessary step but can be done to help with time management.
5. If you have frozen the sample, defrost them on ice. Once thawed you can proceed.
6. The next step requires the disruption and mixing of the biofilm material that is in the Bugbuster® (this may be challenging depending on the sample type, due to viscosity). You can use a 23G needle and a syringe (1-3 ml) and disrupt by repeated passage until the material can be broken up no further.
7. Sonicate the sample using 30% amplitude, 10 second timer pulses with 1 second on and off for 3 seconds. Place the samples on ice until you have processed the samples and are ready for the next step. Clean the probe between samples. (Perhaps consider doing this in batches of up to 20 if you have lots of samples).
8. Incubate the disrupted biomass at 21°C with agitation (300 rpm) for 20 minutes.
9. Centrifuge at 4°C at 17,000 g for 10 minutes – NOTE – if you had a highly mucoid biofilm sample the material here will retain a high level of viscosity. This might mean you are unable to see a pellet.
10. Remove the supernatant (contains the PGA and proteins released from the lysed cells) and place in a labelled fresh tube.
11. You can freeze the samples at -20°C at this point.
12. Quantify the proteins using your preferred method. The Pierce BCA protein assay kit (23227) is compatible with the BugBuster® solution (see manufacturers' instructions).
13. If following the Pierce BCA protein assay kit protocol the 96 well plate is incubated at 37°C for 30 minutes before absorbance is measured at 562 nm on a plate reader. Data is analysed using Microsoft Excel. The measurements are background corrected by subtracting blank values from standard and sample values. A standard curve should be constructed with the values, and the equation of the line used to convert absorbance measurements to protein concentration µg/ml. Quantification of biofilm protein is used to normalise samples for PGA gels and quantification assay.
14. Store the samples at -20°C until required for the next steps.

Analysis of PGA by electrophoresis

Sodium dodecyl sulphate polyacrylamide gel electrophoresis (SDS-PAGE) can be used to separate out extracted biofilm proteins (and PGA) based on size for PGA staining. This PGA profile will be an initial check to ensure PGA is present within samples and can be compared to quantification results. After staining with methylene blue the gel will be dark blue and will require de-staining, which should be continued until dark band(s) become apparent in positive samples and gel imaging becomes possible.

1. SDS-PAGE gels should be prepared according to local procedures. We suggest preparation of a 12% (w/v) lower gel and 7% (w/v) stacking gel (see recipe) that has been cast in a 3:1 ratio. This approach allows PGA to migrate further into the gel while still allowing protein to be visualised).
2. To prepare for loading onto the gel, combine the samples with loading dye and water in Eppendorf tubes. Biofilm extract samples should be adjusted to contain to 5-10 µg protein per sample per well depending on sample type. Note that samples with high PGA content can warp gel wells and necessitate adjustment of protein levels, mix with loading dye and dH_2O as required for a total volume of 25 µl containing 5-10 µg protein.
3. Mix samples well and heat to 99°C±1°C for 5 minutes, after incubation allow to cool and briefly centrifuge tubes to gather the sample and remove liquid from lid.
4. Samples (25 µl) should be loaded onto the acrylamide gel alongside an appropriate molecular weight ladder (e.g. 5 µl, Precision Plus Protein™ Dual Colour Standards (Bio-Rad)).
5. Run gels at 180 V for ~ 1 hour in running buffer (25 mM Tris, 0.192 M glycine, 0.1% SDS (w/v), pH 8.3) until the blue dye front in the sample loading dye has reached the bottom of the gel.
6. Remove gels from tank and glass plates and transfer to a suitable container ready for staining (gels will be fragile so handle with care).
7. Rinse acrylamide gels in H_2O, drain H_2O and soak in Instant Blue® Coomassie protein stain (Abcam) for 1 hr with gentle rocking (50 rpm).
8. After incubation in the Coomassie stain, rinse gels with H_2O and soak in 0.5% (w/v) methylene blue in 3% (v/v) acetic acid for 10-20 minutes with gentle rocking (50 rpm).
9. Wash gels with H_2O until the background non-bound dye is removed and clear bands appear (dispose of diluted methylene blue dye according to local procedures).
10. Image gels using a ChemiDoc Imaging system (VWR) or similar.

Quantification of PGA

For the quantification of PGA, samples are treated (to remove DNA and protein) and diluted. Aliquots (25 µl) of standards (in duplicate) and sample are added to 96 well plates then 275 µl of 25 µM methylene blue is added to each well for a total volume of 300 µl (wells are then mixed avoiding bubbles). The microtiter plates are incubated at room temperature for 10 minutes then the absorbance spectrum measured (400-800 nm) on a plate reader. Data are analysed using Microsoft® Excel®, where measurements are background corrected by subtracting blank values from standard and sample values. Standard curves are constructed from values recorded at 564 nm and 664 nm, and the equation of the line used to convert absorbance measurements to PGA quantities mg/µg of protein.

Part 1: Prepare PGA standard curve

1. To prepare PGA standards for quantification use the purified PGA that was lyophilised and stored in a powdered form at -20°C. See Table 1.
2. Weigh out 10 mg of purified PGA into an Eppendorf tube noting there could be static electricity and you will need to be careful.
3. Add 1 mL Bugbuster® Master Mix (to keep the solution the same as your biofilm samples).
4. Mix gently but thoroughly to completely dissolve the PGA. Suspend well by vortexing.
5. Prepare the standards by adding volumes of required stock to volumes of BugBuster® Master Mix (Table 1).
6. After the preparation of each standard vortex the tube to mix thoroughly before preparing the subsequent standards.
7. Standards should be stored at 4°C until required and vortexed before each use.
8. We recommend using PGA standards from 2 mg/ml and below (higher concentrations have saturation issues).

Part 2: Prepare the samples

1. Normalise the extracted biofilm samples to 10 µg protein per 25 µl (20 µg of sample protein in 50 µL (0.4 µg/µl) final volume (use BugBuster® solution as required).
2. Optional: Treat samples with DNaseI (Qiagen®, 1U/µl) this should be carried out before Proteinase K treatment.
3. Treat samples to remove protein by adding 1µL Proteinase K (Qiagen Proteinase K >600 mAU/ml) and incubate at 56°C for >90 minutes – 4 hours.
4. Pulse centrifuge the tubes to gather the sample at the bottom.

5. Dilute samples to what would be equivalent of 1 µg and 0.1 µg protein per 25 µl diluent.
6. OPTIONAL: You can use 1:10 dilutions of the samples to prepare samples equivalent to 0.04 µg/µl and 0.004 µg/µl (or further should you need). This may be needed to ensure sample absorbance values fall within the standard curve.

Part 3 – Set up the PGA assay plate

1. The assay requires 25 µl volumes of material to be tested (standard or sample) mixed with 275 µl of methylene blue in water (see recipe).
2. Aliquot 25 µl of each standard in duplicate (recommended <2 mg/ml) into a 96 well microtiter plate followed by samples as required, note the plate layout for future reference. Samples may require a dilution series to fall within the standard curve range, see the template for a suggestion of the assay layout.
3. Use a multichannel pipette and add in 275 µL of 25 µM methylene blue in water (see recipe).
4. Mix samples as required avoiding bubbles.
5. Incubate at room temperature (23°C) for 10 minutes.
6. Transfer to a plate reader and measure absorbance across 400-800 nm (in increments of 2 nm), or at the defined wavelengths of 564 nm and/or 664 nm.

Part 4: Calculate the PGA concentration in the sample

1. Generate a standard curve from the data collected.
2. In a spreadsheet blank correct the raw data by subtracting the blank value from each cell (raw reading- blank reading = blank corrected data).
3. Select and organise the blank corrected data. The greatest or maximum peaks formed (when PGA binds methylene blue) should be around 564 nm and 664 nm.
4. Prepare a table with the known values of each PGA standard. For each wavelength (564 nm and 664 nm), copy the blank corrected absorbance readings-corresponding to the appropriate wells (containing the standard)- across into the table.
5. Prepare graphs for each wavelength, use the graph function in Excel to insert an XY scatter plot with the known PGA quantities for the X-axis and the absorbance readings on the Y-axis.
6. Add a trendline and click to display the equation of the line and the R^2 value (to check the fit).
7. The equation of the line should be re-arranged and used to convert absorbance measurements for each sample to PGA quantities mg/µg of protein.

8. Recommended: Prepare a spreadsheet with cut off for standard curve calculate PGA for each dilution prepared and use only wells that fall within standard curve limits.
9. Use data to calculate the mg PGA per 1 mg protein.
